# Transcriptome and metabolome profiling reveal the effects of hormones on current-year shoot growth in Chinese ‘Cuiguan’ pear grafted onto vigorous rootstock ‘Duli’ and dwarf rootstock ‘Quince A’

**DOI:** 10.1186/s12870-024-04858-3

**Published:** 2024-03-05

**Authors:** Zhenxu Liang, Qinghua Wang, Mingde Sun, Ruirui Du, Wanmei Jin, Songzhong Liu

**Affiliations:** Institute of Forestry and Pomology,Beijing Academy of Agriculture and Forestry Sciences, , Beijing Engineering Research Center for Deciduous Fruit Trees, Key Laboratory of Biology and Genetic Improvement of Horticultural Crops (North China), Ministry of Agriculture and Rural Affairs, Beijing, 100093 P.R. China

**Keywords:** Pear, Scion-rootstock, Shoot, Transcriptome, Phytohormones

## Abstract

**Background:**

Dwarf rootstocks have important practical significance for high-density planting in pear orchards. The shoots of ‘Cuiguan’ grafted onto the dwarf rootstock were shorter than those grafted onto the vigorous rootstock. However, the mechanism of shorter shoot formation is not clear.

**Results:**

In this study, the current-year shoot transcriptomes and phytohormone contents of ‘CG‒QA’ (‘Cuiguan’ was grafted onto ‘Quince A’, and ‘Hardy’ was used as interstock) and ‘CG‒DL’ (‘Cuiguan’ was grafted onto ‘Duli’, and ‘Hardy’ was used as interstock) were compared. The transcriptome results showed that a total of 452 differentially expressed genes (DEGs) were identified, including 248 downregulated genes and 204 upregulated genes; the plant hormone signal transduction and zeatin biosynthesis pathways were significantly enriched in the top 20 KEGG enrichment terms. Abscisic acid (ABA) was the most abundant hormone in ‘CG‒QA’ and ‘CG‒DL’; auxin and cytokinin (CTK) were the most diverse hormones; additionally, the contents of ABA, auxin, and CTK in ‘CG‒DL’ were higher than those in ‘CG‒QA’, while the fresh shoot of ‘CG‒QA’ accumulated more gibberellin (GA) and salicylic acid (SA). Metabolome and transcriptome co-analysis identified three key hormone-related DEGs, of which two (*Aldehyde dehydrogenase* gene *ALDH3F1* and *YUCCA2*) were upregulated and one (*Cytokinin oxidase/dehydrogenase* gene *CKX3*) was downregulated.

**Conclusions:**

Based on the results of transcriptomic and metabolomic analysis, we found that auxin and CTK mainly regulated the shoot differences of ‘CG–QA’ and ‘CG–DL’, and other hormones such as ABA, GA, and SA synergistically regulated this process. Three hormone-related genes *ALDH3F1*, *YUCCA2*, and *CKX3* were the key genes contributing to the difference in shoot growth between ‘CG–QA’ and ‘CG–DL’ pear. This research provides new insight into the molecular mechanism underlying shoot shortening after grafted onto dwarf rootstocks.

**Supplementary Information:**

The online version contains supplementary material available at 10.1186/s12870-024-04858-3.

## Background

Grafting is a traditional technique of asexual propagation since antiquity for fruit crop improvement, and has been widely used in many horticultural crops, such as apple [1, 2], pear [3, 4], citrus [5], and grape [6]. Certain rootstocks not only affect the adaptability of fruit trees to the natural environment, but also influence tree growth potential and fruit production efficacy [7]. Numerous studies have demonstrated that dwarf rootstocks could be advantageous for shoot dwarfing to benefit mechanized operations and increase the fruit yield per unit area, and could also enhance fruit crop resistance to abiotic and biotic stresses [8–10]. Although the detailed molecular mechanisms underlying these processes remain incompletely understood and require further research, increasing efforts have found that secondary metabolites play important roles in graft-induced phenotypic variation in anatomy and morphology [11–13]. However, how have the secondary metabolites of the scion grafted onto one rootstock, especially the phytohormones, changed? Which phytohormones contribute more to the formation of scion morphology? In addition, the related genes involved in hormone synthesis and metabolism have yet to be further mined.

Phytohormones as small signaling molecules, play vital roles in plant growth and development processes ranging from seed germination to root extension, flower formation, fruit ripening, as well as abiotic and biotic tolerance [14–16]. More recent research findings have shown that a suitable scion-rootstock connection positively contributes to the translocation of water, minerals, and hormones; in turn, hormones regulate the connection between the scion and rootstock [17]. As for the diversity in shoot forms, it was well established that phytohormones were very important signals regulating the shoot plasticity [18, 19]. Abscisic acid (ABA) which is involved in various plant processes, plays a vital role in biotic and abiotic stress responses, including wounding. During grafting, the expression of wound-related genes is induced by ABA [20]. In addition to ABA, cytokinin (CTK), auxin, gibberellin (GA), and other hormones have also been reported to play vital roles in the formation of vascular tissues after grafting [21]. CTKs are a class of adenine derivatives, such as isopentenyl adenine, and *trans-* and *cis-*zeatin [16]. CTK as one of the classic five phytohormones, affects many processes in plants, including cell division and proliferation in the shoot apical meristem (SAM) [22]. Lowering the cytokinin content or reducing cytokinin signaling results in abbreviating the activity of the SAM [23, 24]. In the CTK catabolism process, cytokinin oxidase/dehydrogenase (CKX) has been reported as a key enzyme regulating CTK levels [25]. In addition, auxin also contributes to the dwarf phenotype of fruit trees by regulating the cell division and cell elongation [26]. Together, these studies provide a reference for research on plant shoot growth.

Pear (*Pyrus* spp.) is one of the major fruit crops in China and accounts for 73.9% of world production, with a production of over 18.9 million tons [27]. ‘Cuiguan’ (*P.* × *pyrifolia*) which possesses strong flavor, few stone cells, and crisp flesh, is widely cultivated in China [28, 29]. Currently, ‘Cuiguan’ is mainly grafted onto the vigorous rootstock in traditional orchards. However, the vigorous planting exhibited high tree height and closed canopy, which would block light to the leaves and consequently reduce fruit yield and quality. It is gratifying that the application of quince (*Cydonia* × *oblonga*) as dwarf rootstock is gradually increasing in modern pear orchards. In addition, because of the different genetic material between ‘Cuiguan’ and quince, interstock ‘Hardy’ is usually selected for overcoming interspecific incompatibility. Although the application of dwarf rootstocks has important practical significance, there are few studies underlying the mechanism of how dwarf rootstocks affect the reproductive and vegetative growth of scions.

As phytohormones play important roles in plant growth and development, we hypothesized that phytohormones contributed to the difference in shoots between vigorous-type scions and dwarf-type scions. In this study, we tested the above hypothesis by using two scion-rootstock combinations, including ‘Cuiguan’/‘Hardy’/‘Duli’ and ‘Cuiguan’/‘Hardy’/‘Quince A’. Then we combined transcriptomic and metabolomic analysis to reveal phytohormone metabolism and identify key hormone-related genes. The results of this study not only provide data for the phytohormone metabolism of ‘Cuiguan’ grafted onto two types of rootstocks, but also provide a theoretical basis for improving the comprehensive utilization of scion-rootstock combinations.

## Results

### Physiological characteristics of planting and shoots

**Fig. 1:**
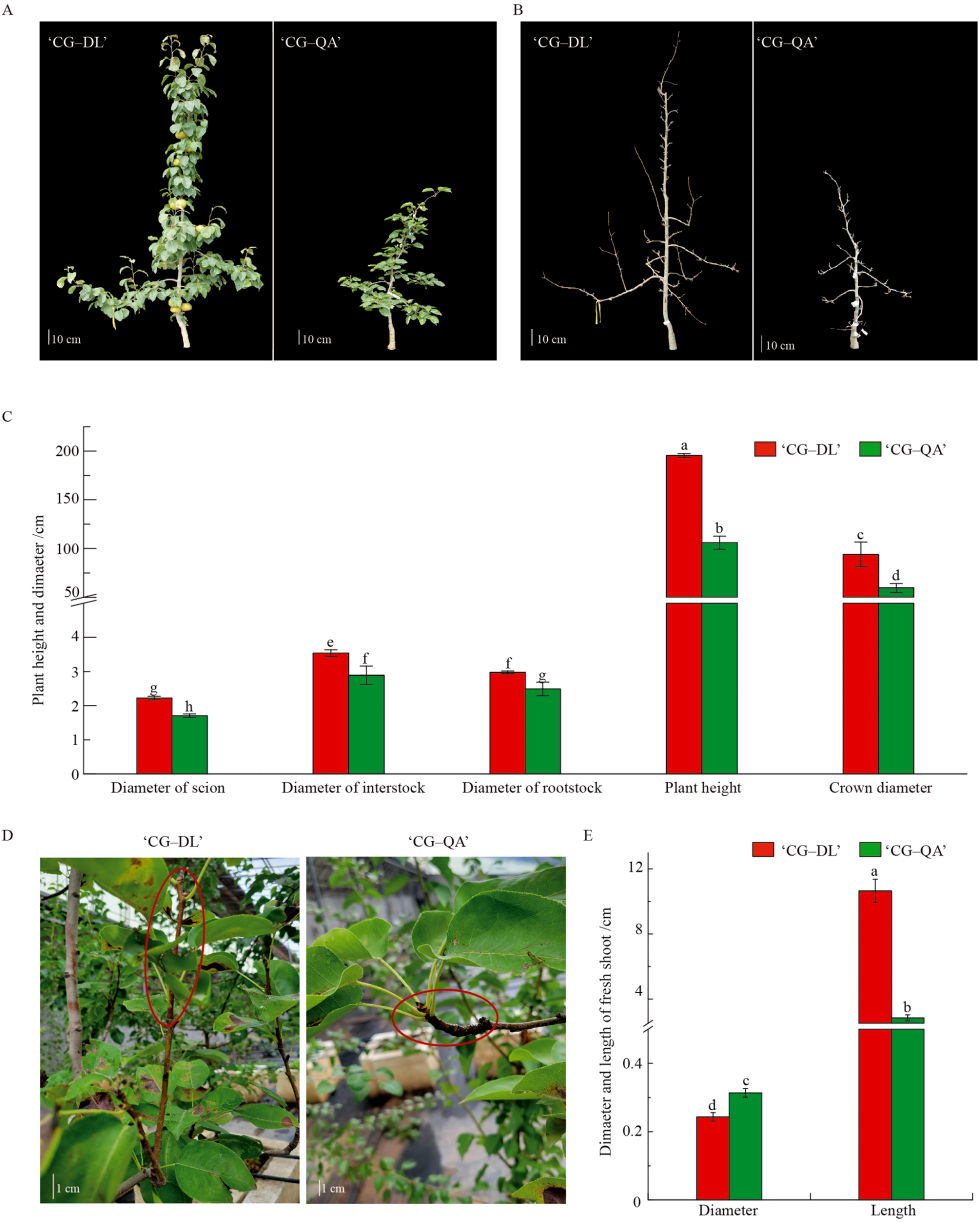
Phenotype of the current-year shoots of ‘CG‒QA’ vs. ‘CG‒DL’. (**A**) Plant phenotype of two grafting combinations in full fruit period. (**B**) Plant phenotype of two grafting combinations during dormancy. (**C**) Physiologic indexes including the diameter of the scion, stock, and crown as well as plant height. (**D**) Phenotype of the current-year shoots of the two grafting groups. (**E**) The diameter and length of the current-year shoot. Lowercase letters, a–h, represent significant differences according to the independent sample t-test (*p* < 0.05) for each sampling time point

The phenotypes of the dwarf-type ‘CG‒QA’ and the vigorous-type ‘CG‒DL’ were shown in Fig. 1A and B. The planting growth vigor of the ‘CG‒DL’ group was more visible, while the ‘CG‒QA’ planting showed obvious dwarf characteristics, such as fine diameter of scion and stock, dwarf stature, and compact crowns (Fig. 1C). Although the diameters of the rootstocks, interstocks, and scions of the two groups were similar at the beginning of the experiment, the three indexes of ‘CG‒QA’ were lower than those of ‘CG‒DL’ after four years of grafting. The plant height of ‘CG‒QA’ was only 54% of ‘CG‒DL’. Interestingly, the current-year shoot length of ‘CG‒QA’ was significantly shorter than that of ‘CG‒DL’ (Fig. 1D and E).

### Transcriptome sequencing data and DEGs analysis

**Fig. 2:**
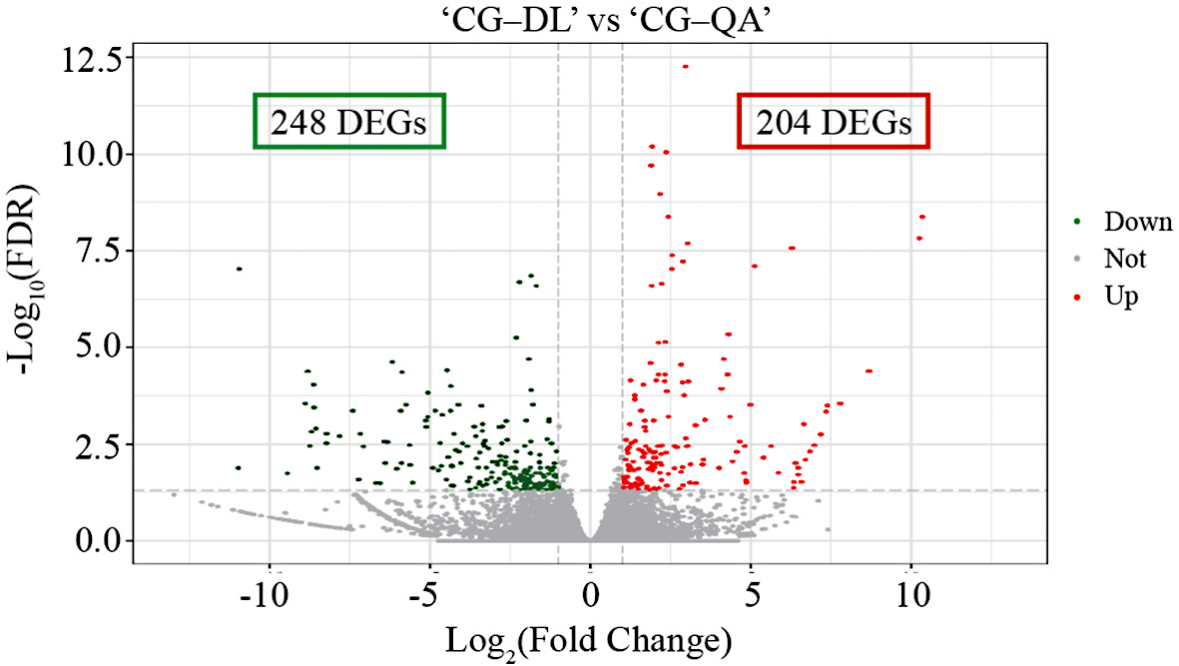
Changes in gene expression levels of ‘Cuiguan’ grafted onto two types of rootstocks

Six cDNA libraries were constructed from the current-year shoots of ‘Cuiguan’, which were respectively grafted onto different rootstocks including ‘Hardy’ + ‘Duli’ and ‘Hardy’ + ‘Quince A’. A total of 37.09 Gb clean data were obtained and the clean data of each sample reached more than 5.78 Gb, and the average GC content was 48.20%. The Q20 and Q30 base percentages were more than 98.00% and 93.81%, respectively. The individual data for six cDNA libraries were shown in Table 1. A total of 452 DEGs were identified, including 248 downregulated genes and 204 upregulated genes (Fig. 2).

**Table 1:** Summary of transcriptome sequencing data of sample ‘CG‒DL’ and ‘CG‒QA’

Sample ID	Scion	Interstock	Rootstock	Clean reads	Clean data (bp)	GC (%)	Q20 (%)	Q30 (%)
‘CG‒DL’1	‘Cuiguan’	‘Hardy’	‘Duli’	19,284,468	5,785,340,400	48.67	98.06	93.98
‘CG‒DL’2	‘Cuiguan’	‘Hardy’	‘Duli’	19,306,196	5,791,858,800	49.03	98.32	94.68
‘CG‒DL’3	‘Cuiguan’	‘Hardy’	‘Duli’	21,043,047	6,312,914,100	48.07	98.10	94.13
‘CG‒QA’1	‘Cuiguan’	‘Hardy’	‘Quince A’	22,520,421	6,756,126,300	47.83	98.00	93.81
‘CG‒QA’2	‘Cuiguan’	‘Hardy’	‘Quince A’	20,588,925	6,176,677,500	47.74	98.35	94.78
‘CG‒QA’3	‘Cuiguan’	‘Hardy’	‘Quince A’	20,884,692	6,265,407,600	47.87	98.09	94.14

### GO and KEGG annotation of DEGs in ‘Cuiguan’ grafted onto different rootstocks

**Fig. 3:**
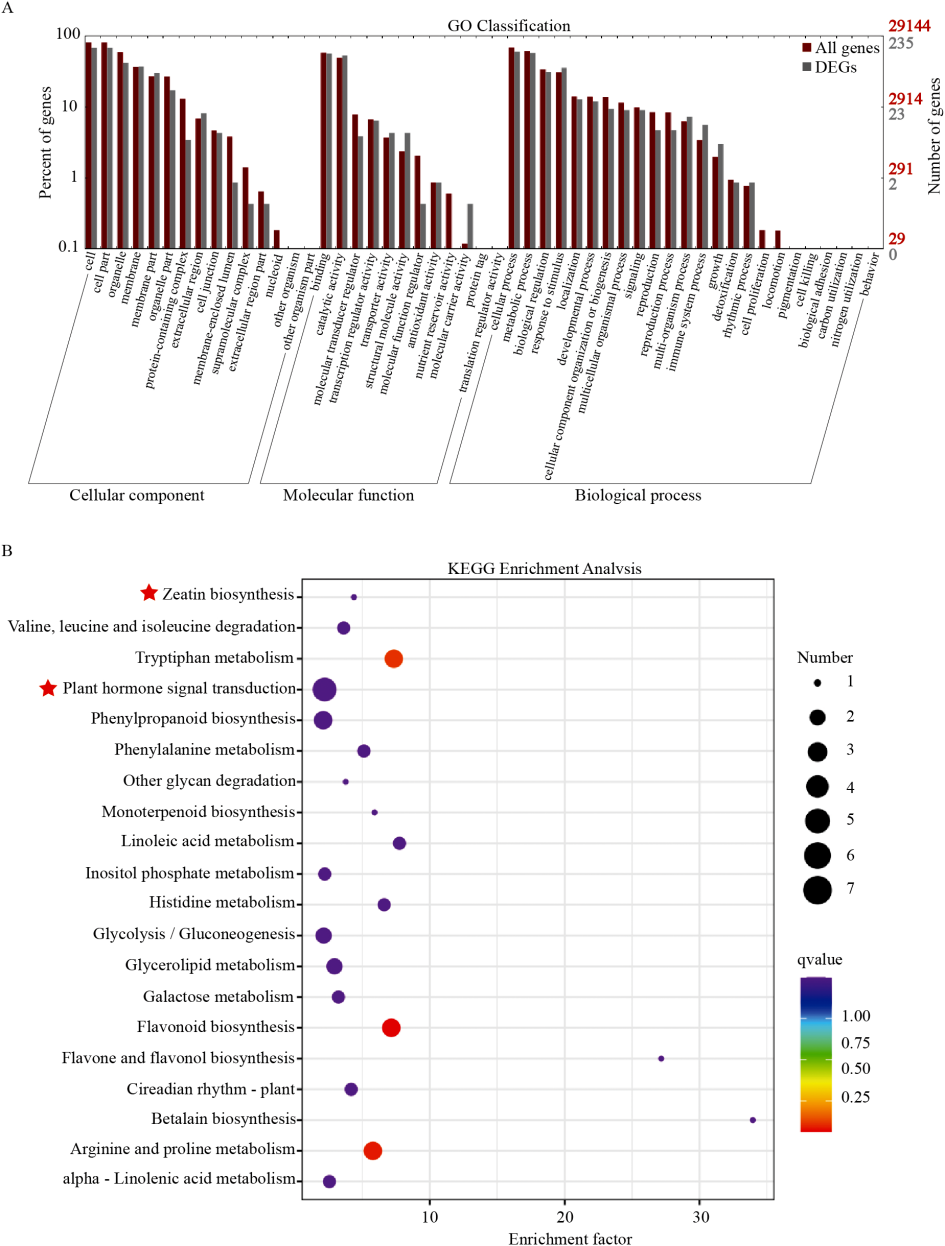
GO and KEGG enrichment analysis of DEGs. (**A**) GO enrichment of DEGs. (**B**) KEGG enrichment analysis of DEGs. We have obtained appropriate copyright permission to use the KEGG image

GO enrichment analysis of the DEGs was implemented by the cluster Profiler R package, and the functional categories included cellular component, molecular function, and biological process. According to the GO enrichment results, a total of 235 DEGs were categorized into 37 functional groups (Fig. 3A). Among the cellular component functional groups, the ‘cell’ and ‘cell part’ were the two most abundant groups. In the molecular function and biological progress categories, the largest groups were binding and cellular process, respectively.

To explore the metabolic pathways, Kyoto Encyclopedia of Genes and Genomes (KEGG) pathway analysis was further used in DEGs analysis. The top 20 KEGG enrichment terms were listed in Fig. 3B, with significantly enriched pathways putatively identified as ‘plant hormone signal transduction’. Simultaneously, the ‘Tryptophan metabolism’, ‘Phenylpropanoid biosynthesis’, ‘Flavonoid biosynthesis’, and ‘Arginine and proline metabolism’ were also significantly enriched in the KEGG pathway. Interestingly, we found that the ‘zeatin biosynthesis’ was highly enriched in the ‘CG‒DL’ vs ‘CG‒QA’ comparison group. The above results indicated that hormone synthesis and metabolism played important roles in the difference in shoots of the ‘CG‒DL’ vs ‘CG‒QA’ group.

### Screening of differentially expressed genes related to hormones

**Fig. 4:**
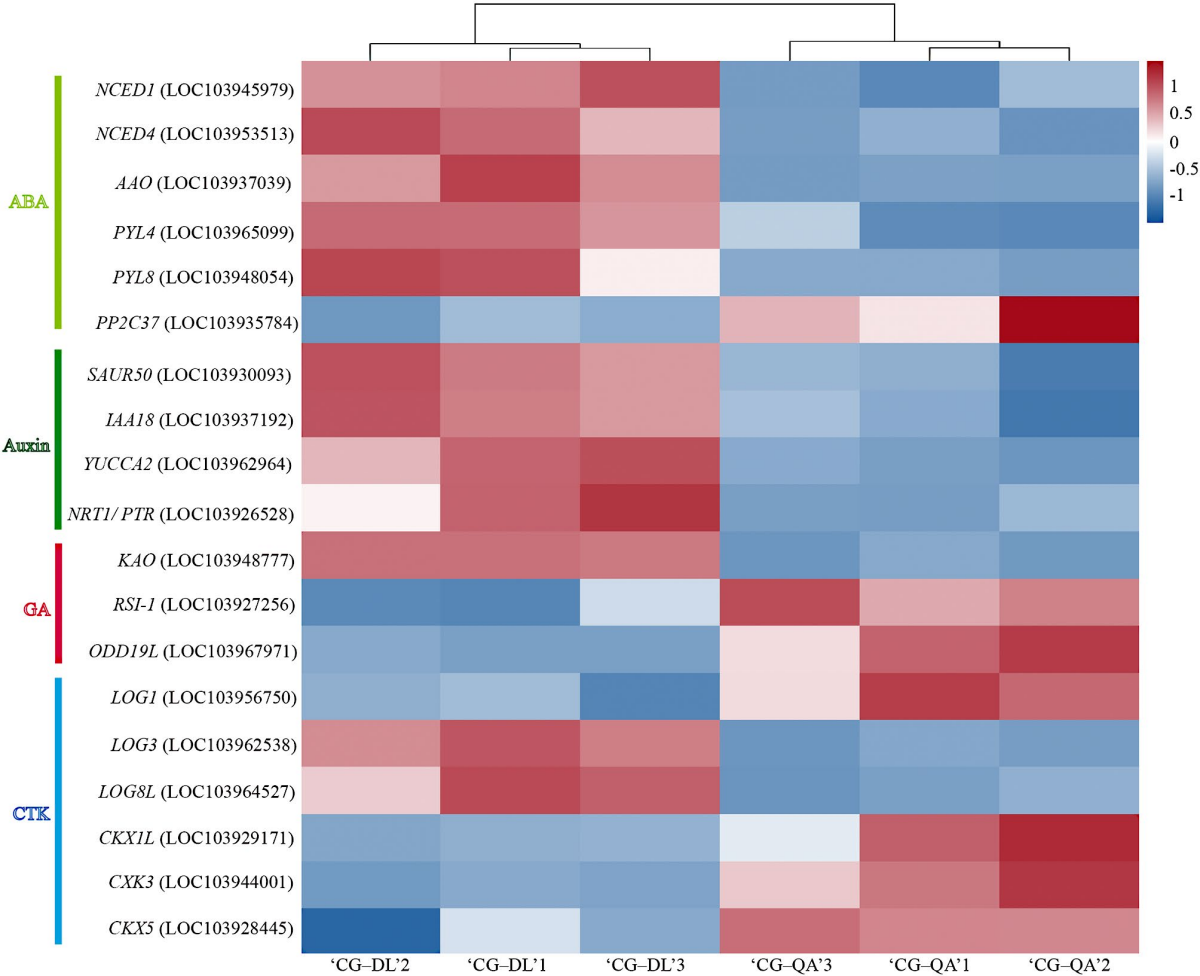
Relative expression of hormone-related genes

Based on our transcriptome results, a total of 19 DEGs were predicted to be associated with plant hormones. As shown in Fig. 4, these DEGs were divided into four groups, including ABA-related genes (*9-cis-epoxycarotenoid dioxygenase* gene *NECD1*, *NCED4*, *ABA-aldehyde oxidase* gene *AAO*, *pyrabactin resistance 1-like* gene *PYL4*, *PYL8*, and *protein phosphatase 2 C* gene *PP2C37*), auxin-related genes (*small auxin-up RNA* gene *SAUR50*, *IAA18*, *YUCCA2*, and *nitrate absorption and transport* gene *NRT1/PTR*), GA-related genes (*ent-kaurenoic acid oxidase* gene *KAO*, *lateral root initiation* gene *RSI-1*, and *2-oxoglutarate-dependent dioxygenase* gene *ODD19L*), and CTK-related genes (*cytokinin riboside 5’-monophosphate phosphoribohydrolase* gene *LOG1*, *LOG3*, *LOG8L*, *cytokinin oxidase/dehydrogenase* gene *CKX1L*, *CKX3*, and *CKX5*). The expression levels of numerous hormone-related DEGs showed differences between the two groups. Notably, compared with ‘CG‒DL’, most ABA-related genes exhibited lower expression in ‘CG‒QA’, except for phosphatase *PP2C37*. The expression levels of the four auxin-related genes were significantly higher in ‘CG‒DL’ than in ‘CG‒QA’. The *ent*-kaurenoic acid oxidase gene *KAO* and 2-oxoglutarate-dependent dioxygenase gene *ODD19L* exhibited high expression levels in ‘CG‒QA’, and the *RSI-1* had an opposite expression pattern. Among these CTK-related DEGs, the three *LOG* genes encoding CTK activating enzymes exhibited different expression patterns. The expression level of *LOG1* was higher in ‘CG‒QA’, while the *LOG3* and *LOG8L* genes showed the opposite pattern. A total of the three *cytokinin oxidase/dehydrogenase* gene *CKXs* showed high expression levels in ‘CG‒QA’ group. The above results suggested that hormone-related genes could be involved in the formation of short shoots in ‘CG‒DL’.

### Determination of the phytohormones of ‘CG‒DL’ and ‘CG‒QA’

**Fig. 5:**
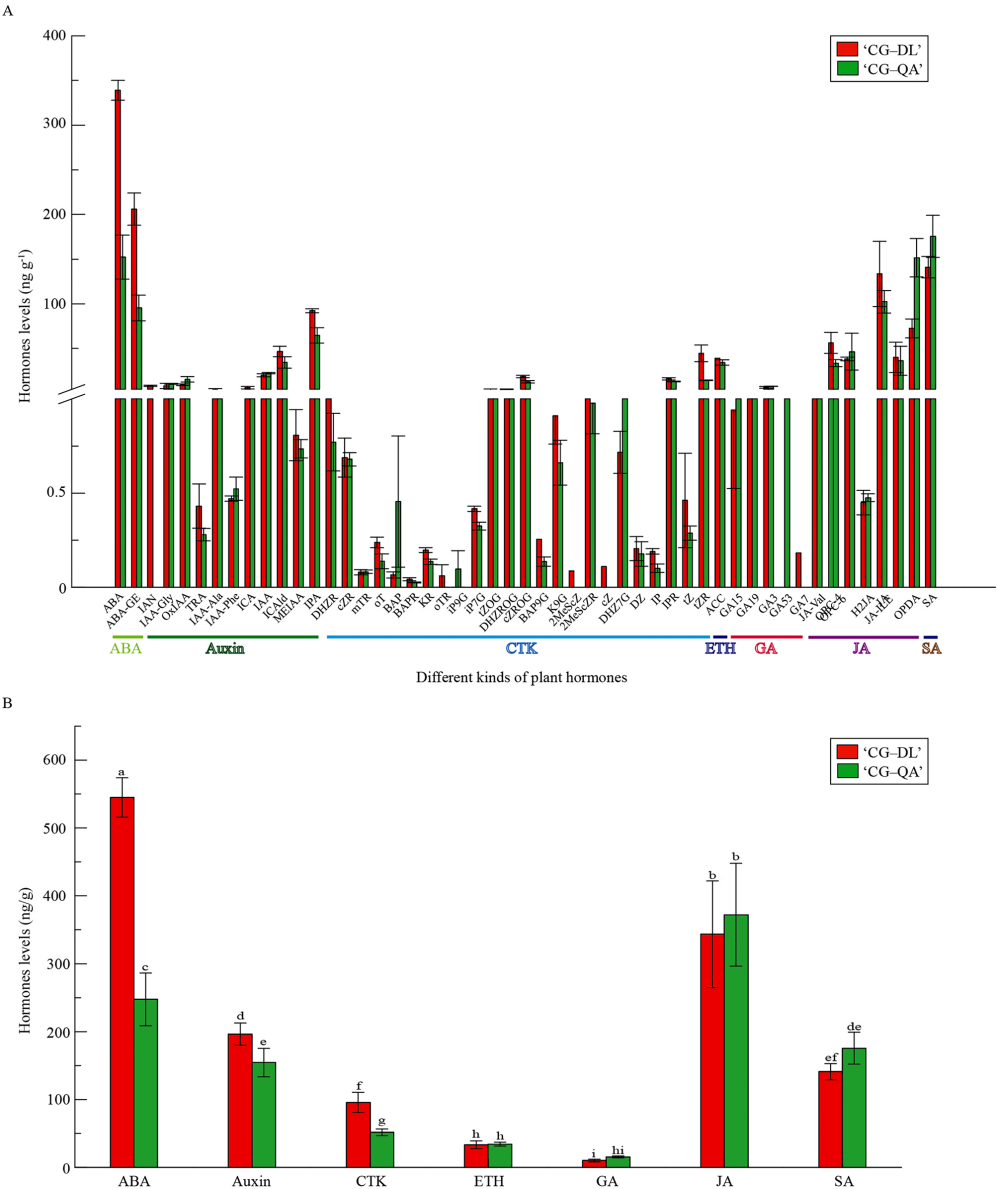
Histogram analysis of differential hormones in ‘CG‒QA’ and ‘CG‒DL’. (**A**) Fifty hormone components in ‘CG‒QA’ and ‘CG‒DL’. (**B**) Seven categories of endogenous hormone content in ‘CG‒DL’ and ‘CG‒QA’. Lowercase letters, a–i, represent significant differences according to the independent sample t-test (*p* < 0.05) for each sampling time point

Plant shoots are derived from the shoot apical meristem (SAM), which is regulated by many exogenous and endogenous factors, such as phytohormones. To investigate the effect of phytohormones on the current-year shoot formation of ‘Cuiguan’, we determined the contents of different hormones in the shoots of ‘CG‒DL’ and ‘CG‒QA’ (Fig. 5). A total of 45 hormone components were detected in the ‘CG‒QA’ (Fig. 5A). The types of CTKs were the most numerous (20) in ‘CG‒QA’, followed by auxin (10), JA (7), GA (4), ABA (2), ETH (1), and SA (1). A total of 50 hormone components were identified in the ‘CG‒DL’ group, including 23 CTKs, 11 auxins, 7 JAs, 4 GAs, 2 ABAs, 1 ETH, and 1 SA (Fig. 5A). Among the above hormones, the total contents of ABA were the highest in both groups ‘CG‒DL’ and ‘CG‒QA’, with contents of 545 ng g^− 1^ and 248 ng g^− 1^, respectively (Table S1). Notably, the contents of ABA, auxin, and CTK in fresh shoots of ‘CG‒QA’ were lower than those in ‘CG‒DL’, while the fresh shoots of ‘CG‒QA’ contained more GA and SA, and there was no significant difference in the contents of ETH and GA between ‘CG‒DL’ and ‘CG‒QA’ (Fig. 5B). Since CTK and auxin played important roles in the elongation of shoot, we were more interested in these two hormones. In addition, indole-3-pyruvic acid (IPA) and *trans*-Zeatin-riboside (*t*ZR) were the most abundant auxin and CTK in ‘CG‒DL’ and ‘CG‒QA’, respectively. The above results suggested that CTK and auxin contributed to the difference in shoot formation between ‘CG‒DL’ and ‘CG‒QA’, and other hormones such as ABA, GA, and SA also synergistically regulated this process.

### Combined transcriptome and metabolome analyses

**Fig. 6:**
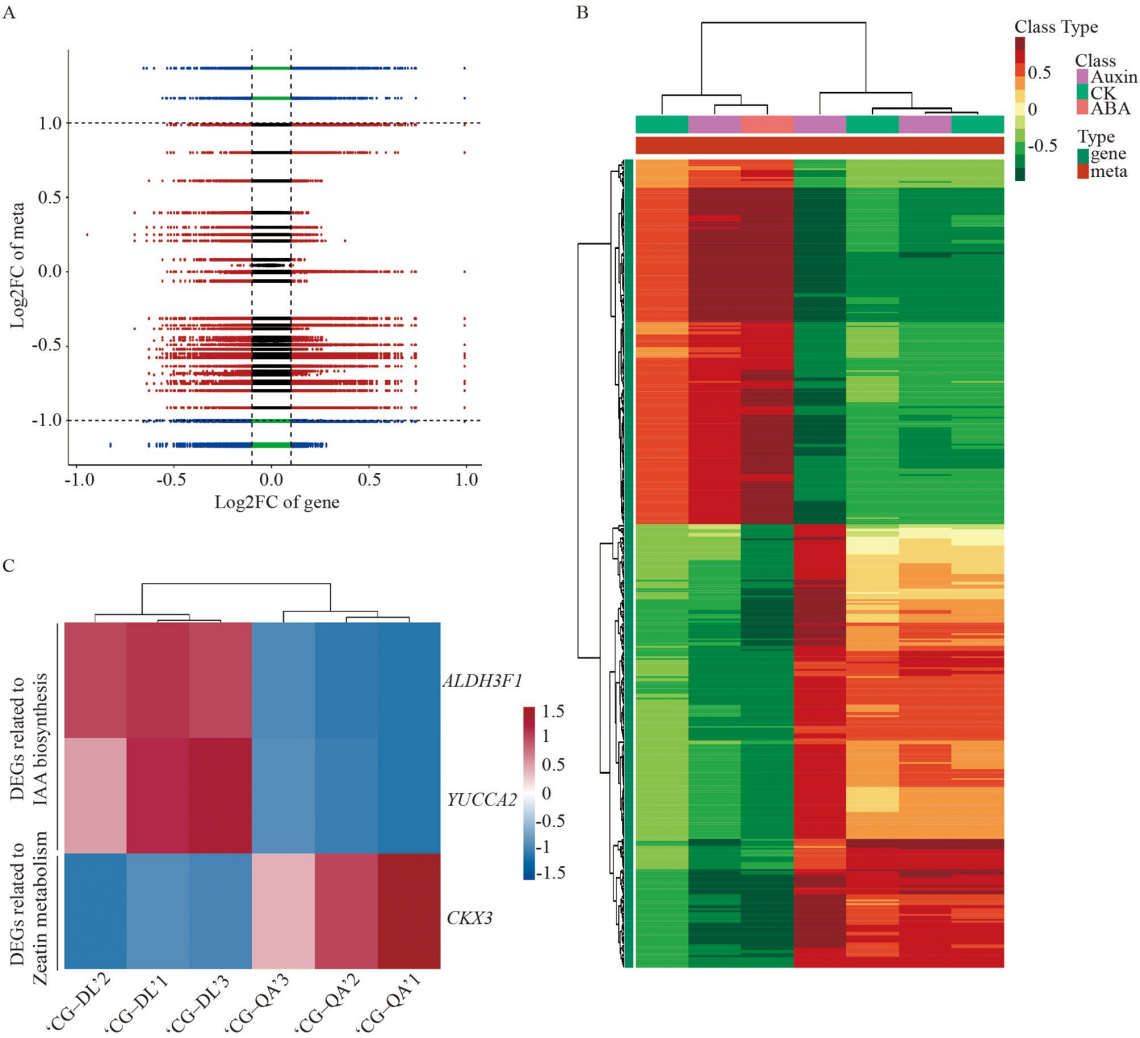
Integrated analysis of the DEGs and DAMs between ‘CG‒DL’ vs. ‘CG‒QA’. (**A**) Correlation analysis nine quadrant chart. Blue dots indicate combined DEGs and DAMs; green and red dots indicate DAMs with unchanged genes or DEGs with unchanged metabolites; black dots indicate unchanged genes and metabolites. (**B**) Heatmaps of the correlation coefficient clusters. Green indicates genes; orange indicates metabolites. (**C**) Screening of candidate key genes associated with hormone synthesis and metabolism based on joint analysis. Red indicates high expression; blue indicates low expression

To further investigate the mechanism of the difference in shoot formation between ‘CG‒DL’ and ‘CG‒QA’ at the hormone and molecular levels, a detailed analysis of transcriptome and metabolomic data was conducted. Figure 6A showed the Pearson correlation coefficients (PCC) for the nine quadrants. The patterns of the DEGs and differentially accumulated metabolites (DAMs) were consistent in the third and seventh quadrants, and the regulation of genes and metabolites had a positive correlation. The DAMs and DEGs with PCCs higher than 0.8 were further selected and described as a heatmap (Fig. 6B). According to the above results, as shown in Fig. 6C, we identified three DEGs involved in the above two pathways, including *ALDH3F1* (LOC103938024), *YUCCA2* (LOC103962964), and *CKX3* (LOC103944001). Our results showed that the expression levels of *ALDH3F1* and *YUCCA2* were higher in ‘CG‒DL’, and *CKX3* gene expression showed the opposite pattern.

### RT-qPCR validation of transcriptome data

**Fig. 7:**
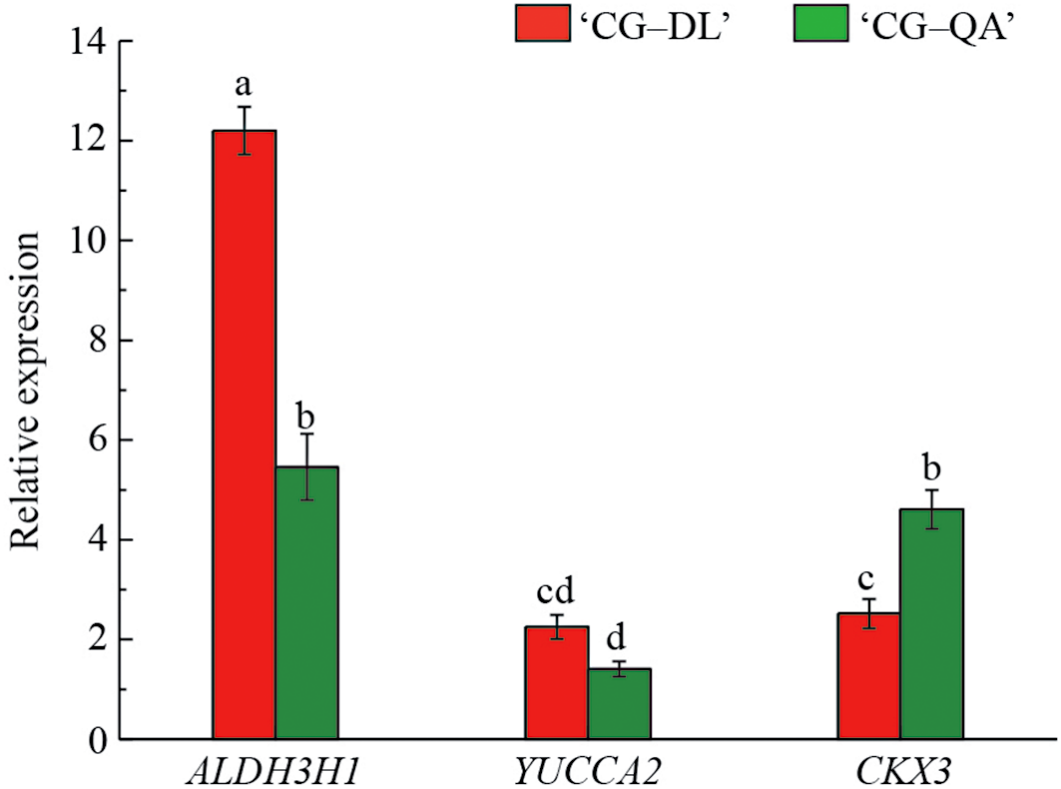
RT-qPCR validation of the relative expression levels of three selected DEGs from ‘CG‒DL’ and ‘CG‒QA’. Lowercase letters, a–d, represent significant differences according to the independent sample t-test (*p* < 0.05) for each sampling time point

To validate the reliability of the transcriptome data of the above three DEGs, we further verified the expression levels of these genes by RT-qPCR. The gene-specific primers used in this analysis were listed in Supplementary Table 2. The expression profiles of *ALDH3H1*, *YUCCA2*, and *CKX3* were corresponding to the transcriptome results (Fig. 7). Thus, it can be inferred that the RNA-seq data were reliable.

## Discussion

Grafting as a well-developed practice has been widely used in horticultural production. The aim of scions grafted onto dwarf rootstocks is to weaken tree vigor and increase planting density [8]. In this study, grafting ‘Cuiguan’ onto the dwarf rootstock ‘Quince A’ not only influenced planting height, but also caused the current-year shoots to become shorter (Fig. 1). In view of the above phenomenon, we carried out research at the physiological and molecular levels.

The effects of vigorous rootstock and dwarf rootstock on scion are very distinct. In vigorous rootstock orchards, fruit trees exhibit significant vigor characteristics, such as tall planting, strong shoots, and high drought resistance [2]. On the contrary, the vegetative growth of fruit trees grafted onto dwarf rootstock was inhibited. ‘Duli’ and quince are widely used in pear as vigorous rootstocks and dwarf rootstocks, respectively. In our current study, ‘Cuiguan’ grafted onto ‘Quince A’ showed dwarf phenotypes, one of which was shorter shoots (Fig. 1D and E). In addition, Cong et al. found that another dwarf rootstock ‘Yunnan’ quince could significantly promote pear flower bud formation compared with ‘Duli’ [30]. Our results together with other reports showed that the dwarf rootstock played an important role in the reproductive and vegetative growth of fruit trees. We next used RNA-seq to compare the ‘Cuiguan’ grafted onto two rootstock types at the gene level. A total of 452 DEGs were identified in our results, including 248 downregulated genes and 204 upregulated genes (Fig. 2). In summary, at both the physiological and genetic levels, the ‘Cuiguan’ plants grafted onto vigorous and dwarf rootstocks were significantly different. Because excessive vegetative growth always consumes many nutrients and leads to yield reduction, it is particularly important to maintain the dynamic balance between vegetative growth and reproductive growth of fruit trees [8, 31]. Dwarf rootstock and high-density planting are important development trends in modern fruit orchards [26, 32]. However, the mechanism of the effects of dwarf rootstocks on scions is not clear, and it is necessary to carry out corresponding research.

Recently, considerable efforts have been made to explore the physiological and molecular mechanisms by which rootstocks affecting scions. For instance, the dwarf pear has fewer xylem cells, which reduces the transport efficiency of water [33]. Furthermore, important progress has also been made in the study of the effects of hormones on rootstock-scion combinations. The dwarf phenotype is closely correlated with a reduction in cell division and elongation, which are induced by phytohormones, such as auxin, CTK, ABA, and GA [32, 34, 35]. Based on our RNA-seq results, the plant hormone signal transduction and zeatin biosynthesis were significantly enriched in the KEGG pathway (Fig. 3B). Then we screened out 19 hormone-related DEGs, including ABA-related DEGs, auxin-related DEGs, GA-related DEGs, and CTK-related DEGs (Fig. 4). Therefore, we hypothesized that the differences in the length of shoots may be related to phytohormones. To verify this hypothesis, we determined the hormone contents in current-year shoots of ‘Cuiguan’ grafted onto two rootstock types. The results showed that regardless of the type or content of hormones, there were many differences between vigorous-type and dwarf-type ‘Cuiguan’ pear. (Fig. 5 and Table S1). Auxin, which is considered to be one of the most important hormones related to dwarf-type planting, is synthesized in the shoot apical meristem (SAM) and is transported downward to plant roots [36, 37]. Previous study found that low levels of IAA limited the growth of the apple tree canopy [38]. It was well established that auxin as an important factor regulated second messenger into axillary buds [39]. And this process leaded to the difference in the activity of buds, which results in diverse shoots [40]. In this study, not only the contents but also the numbers of auxin in vigorous-type pears were greater than those in dwarf-type pears (Fig. 5). Considering this result, we speculated that auxin might play a pivotal role in the morphology of the two types of shoots. As one of the important hormones involved in cell division, CTK is also important for plant dwarfing. Dwarf-type trees exhibit a poor vascular cambium, which leads to low upward transportation of CTK [32]. Similar to auxin, CTK accumulated more in the shoots of the vigorous-type pear, especially *trans*-Zeatin-riboside (*t*-ZR). The opposite transport directions of CTK and auxin play important roles in the formation of dwarf morphology by interacting with each other. Furthermore, current studies found that ABA [41], BR [42], and GA [43] also influenced the growth and development of scions by affecting the formation of vascular tissue at the grafting union. In this study, the contents of GA and SA were much higher in ‘CG‒QA’, while there was no obvious difference of both JA and ETH contents in ‘CG‒DL’ and ‘CG‒QA’. Previous studies showed that ABA as a growth inhibitory hormone had a negative effect on plant growth [44, 45]. However, these studies mainly focused on the effects of exogenous ABA. In this study, endogenous ABA determined was more accumulated in ‘CG‒DL’. It is speculated that endogenous ABA may act as a limiting factor to maintain the dynamic balance of hormones, thereby preventing excessive growth of current-year shoots. The above results demonstrated that the difference in shoots of ‘CG‒DL’ and ‘CG‒QA’ pears was mainly caused by phytohormones CTK and auxin, while ABA, GA, and SA played synergistic regulatory roles.

To further explore the mechanism of shoot formation in dwarf-type ‘CG‒QA’ pear at the gene level, we subsequently screened three key DEGs with transcriptome and metabolome combined analysis in ‘CG‒DL’ (control) vs. ‘CG‒QA’, of which two (*ALDH3F1* and *YUCCA2*) were downregulated and one (*CKX3*) were upregulated (Fig. 6C). *YUCCAs* encoding key enzymes for converting indole-3-pyruvate into IAA play pivotal roles in auxin biosynthesis pathways [46]. Therefore, we hypothesized that the *YUCCA2* promoted the synthesis of IAA, thereby playing an important role in current-year shoot growth in ‘CG‒DL’. Chen et al. found a similar conclusion that the Arabidopsis quintuple mutants *yucca1/2/4/6* have severely disturbed vascular strands, suggesting that the *YUCCAs* contributed to the development of vascular tissue by affecting the synthesis of auxin biosynthesis [47]. ALDH is another important gene for the biosynthesis of auxin, and it convert indole-3-acetylaldehyde into IAA [48]. Furthermore, CTK is also considered an important hormone participating in the dwarfism mechanism. The *Arabidopsis ckx3ckx5* double mutant formed a thicker shoot, which was 15% larger in diameter than the wild type, and the mutant line exhibited large floral meristems by increasing the level of endogenous CTKs [49]. Based on our research and previous reports, these results indicated that these three genes were related to the formation of short fresh shoots in ‘CG‒QA’ pear by directly participating in the synthesis and metabolism of hormones.

## Conclusion

In summary, the shoots of ‘Cuiguan’ showed differences in length after grafted onto the vigorous rootstock ‘Duli’ and dwarf rootstock ‘Quince A’. We analyzed the above phenomenon by combining physiological, transcriptome, and metabolite analysis. We identified nineteen hormone-related DEGs and fifty kinds of hormones in ‘CG‒DL’ and ‘CG‒QA’ pear. Based on the transcriptome and metabolite co-analysis, auxin and CTK mainly contributed to the difference in shoots of ‘CG‒DL’ and ‘CG‒QA’, accompanied by the synergistic regulation of ABA, GA, and SA. Additionally, *ALDH3F1*, *YUCCA2*, and *CKX3* were three key genes participating in synthesis and metabolism of hormones in fresh shoots of ‘CG‒DL’ and ‘CG‒QA’. These results provide a theoretical basis for the molecular mechanism underlying shoot shortening after grafted onto dwarf rootstocks. And our findings were expected to provide new insights for the effects of dwarf rootstocks on pear scions.

## Materials and methods

### Plant materials

In spring 2017, ‘Cuiguan’ were grafted onto the vigorous rootstock ‘Duli’ and dwarf rootstock ‘Quince A’ with ‘Hardy’ as an interstock. Thus, two grafting combinations were constructed as ‘Cuiguan’/’Hardy’/‘Quince A’ (named ‘CG‒QA’) and ‘Cuiguan’/’Hardy’/‘Duli’ (named ‘CG‒DL’). The seedings with robust growth and developed roots were planted in glass pots (80 cm × 80 cm × 80 cm), and the pots were placed in a glasshouse at the Beijing Academy of Agriculture and Forestry Science, Beijing, PR China (39°56’ N, 116°56′ E) in spring of 2018, and subjected to normal agricultural management. In early June 2021, nine plantings with the same growth of two combinations were respectively selected as the experimental materials of this study. Current-year shoots in the middle of ‘Cuiguan’ tree were sampled and instantly frozen in liquid nitrogen for RNA-sequence (RNA-seq) analysis. Every three grafted plantings were combined as one biological repeat, and three biological replicates were performed for subsequent studies.

### RNA extraction, cDNA library construction, and sequencing of fresh stems

Current-year shoots of different varieties were prepared for RNA-seq. Total RNA was extracted using a HiPure HP Plant RNA Mini Kit (Magen, Guangdong, China). RNA purity and concentration were tested with a NanoDrop 2000 spectrophotometer (Thermo Scientific, USA). RNA integrity was assessed using an RNA Nano 6000 Assay Kit of the Agilent Bioanalyzer 2100 system (Agilent Technologies, CA, USA). Sequencing libraries were constructed using NEBNextR UltraTM Directional RNA Library Prep Kit for IlluminaR (NEB, USA) and sequenced on an Illumina Hiseq Xten platform by Genepioneer Biotechnologies (Nanjing, China). The RNA-seq was performed with three repeats.

### Differential expression analysis and functional enrichment

Gene expression levels were estimated by fragments per kilobase of transcript per million fragments mapped (FPKM). Afterwards, differentially expressed genes (DEGs) of two the varieties were identified using the DESeq R package. Specifically, genes with an adjusted P value < 0.01 and |log2-fold change| >2 were assigned as differentially expressed. In addition, GO and KEGG pathway enrichment analysis were performed on the annotated DEGs [50]. GO was implemented by the cluster Profiler R package and KOBAS software was used to test the statistical enrichment of differentially expressed genes in KEGG pathways.

### Extraction of phytohormones from fresh stems and LC–MS analysis

Current-year shoots of the two varieties were harvested, weighed, immediately frozen in liquid nitrogen, and stored at − 80 °C until needed. Three biological replicates were sampled per variety. Fifty milligrams of fresh shoots were ground into powder, and extracted with 1 mL methanol/water/formic acid (15:4:1, V/V/V). The combined extracts were evaporated to dryness under nitrogen gas stream, reconstituted in 100 µL 80% methanol (V/V), and filtered through a 0.22 μm filter for further UPLC‒MS analysis.

A UPLC‒ESI‒MS/MS system (UPLC, ExionLC™ AD; MS, Applied Biosystems 6500 Triple Quadrupole) with a Waters ACQUITY UPLC HSS T3 C18 (100 mm×2.1 mm i.d.1.8 μm) was used to analyze the phytohormone components. The UPLC conditions were as follows: solvent system, water with 0.04% acetic acid (A), acetonitrile with 0.04% acetic acid (B); gradient program, started at 5% B (0–1 min), increased to 95% B (1–8 min), 95% B (8–9 min), finally ramped back to 5% B (9.1–12 min); flow rate, 0.35 mL/min; temperature, 40 °C; injection volume: 2 µL. An AB 6500 + QTRAP® LC‒MS/MS System, equipped with an ESI Turbo Ion-Spray interface, was applied for assay development. The ESI source operation parameters were as follows: ion source, turbo spray; source temperature 550 °C; ion spray voltage (IS): + 5500 V (Positive), − 4500 V(Negative); curtain gas (CUR) was set at 35.0 psi; and DP and CE for individual MRM transitions were performed with further DP and CE optimization. A specific set of MRM transitions was monitored for each period according to the phytohormones eluted within this period.

### Combined analysis of the metabolome and transcriptome

According to the metabolome and transcriptome data, we determined the correlation between metabolites and genes by using the Pearson correlation algorithm (PCA). The DEGs and DAMs of the “phytohormones” in the two varieties were examined based on the above results, and their common pathway information was mapped to KEGG.

### Reverse transcription and quantitative real-time PCR (RT-qPCR) analysis

An RT-qPCR assay was performed using the Roche LightCycler® 96 Real-Time PCR System (Roche Diagnostics GmbH, Mannheim, Germany) and a TransStart® Top Green qPCR SuperMix kit (TransGen Biotech, Beijing, China). The reactions (20 µL) contained 10 µL of Top Green qPCR SuperMix, 0.4 µL of 10 µM forward primer, 0.4 µL of 10 µM reverse primer, and 1 µg cDNA template. qPCR conditions were as follows: an initial denaturation at 95 °C for 30 s, followed by 45 cycles of denaturation at 95 °C for 5 s, annealing at 60 °C for 15 s, extension at 72 °C for 10 s, and a final extension at 72 °C for 3 min. The relative expression level of the genes was calculated using the 2^−ΔΔCt^ method. The primer sequences for RT-qPCR analysis are listed in Supplementary Table 2.

### Statistical analysis

Statistical analyses of significant differences were performed using SPSS software (SPSS Inc., Chicago, IL, USA). Graphs were prepared using Origin 8.1 (Microcal Software, America) and Adobe Illustrator (CC 2017; Adobe Inc., San Jose, America). All the data are presented as the mean ± standard deviation (SD) of 3 independent biological replications.

### Electronic supplementary material

Below is the link to the electronic supplementary material.

Supplementary Material 1

## Data Availability

The data that support the findings of this study have been deposited into CNGB Sequence Archive (CNSA) of the China National GeneBank DataBase (CNGBdb) with accession number CNP0004817. The web link is https://db.cngb.org/search/project/CNP0004817/.

## Declarations

b) The shoots of ‘Cuiguan’ were harvested from one glasshouse in Beijing Academy of Agriculture and Forestry Science, Beijing, PR China. We have permission to collect the materials.

## Abbreviations

‘CG‒DL: Cuiguan/Hardy/Duli
CG‒QA: Cuiguan/Hardy/ Quince A
ABA: Abscisic acid
CTK: Cytokinin
GA: Gibberellin
ETH: Ethylene
JA: Jasmonic acid
KAO: *ent*-kaurenoic acid oxidase
ODD: 2-oxoglutarate-dependent dioxygenase
CKX: Cytokinin oxidase/dehydrogenase
PCC: Pearson correlation coefficients
DEGs: Different expression genes
DAMs: Differentially accumulated metabolites
