# Supplementary material for: Transcriptome and metabolome profiling reveal the effects of hormones on current-year shoot growth in Chinese ‘Cuiguan’ pear grafted onto vigorous rootstock ‘Duli’ and dwarf rootstock ‘Quince A’
Source: BMC Plant Biol. 2024 Mar 5;24:169. doi: 10.1186/s12870-024-04858-3 (PMC10913655; doi:10.1186/s12870-024-04858-3)
Supplement: Supplementary file 1 — Supplementary Material 1 [file 12870_2024_4858_MOESM1_ESM.docx]

Supplemental Table 1 Composition and contents of phytohormones identified in fresh shoot of the ‘CG‒DL’ and ‘CG‒QA’

| Class | Index | Compounds (ng g^-1^ FW) | ‘CG‒DL’-1 | ‘CG‒DL’-2 | ‘CG‒DL’-3 | ‘CG‒QA’-1 | ‘CG‒QA’-2 | ‘CG‒QA’-3 |
| --- | --- | --- | --- | --- | --- | --- | --- | --- |
| ABA | ABA | Abscisic acid | 335 | 354 | 328 | 165 | 174 | 118 |
| ABA | ABA-GE | ABA-glucosyl ester | 223 | 221 | 214 | 78 | 95 | 113 |
| Auxin | IAN | 3-Indoleacetonitrile | 7.33 | 9.24 | 7.95 | ND | ND | ND |
| Auxin | IAA-Gly | Indole-3-acetyl glycine | 11.4 | 4.46 | 8.22 | 11 | 9.88 | 10.35 |
| Auxin | OxIAA | 2-oxindole-3-acetic acid | 7.98 | 9.61 | 10.15 | 12.8 | 19.6 | 13.68 |
| Auxin | TRA | Tryptamine | 0.544 | 0.27 | 0.48 | 0.234 | 0.312 | 0.29 |
| Auxin | IAA-Ala | N-(3-Indolylacetyl)-L-alanine | 3.98 | 5.19 | 3.75 | 2.01 | 2.56 | 2.18 |
| Auxin | IAA-Phe | phenylalanine | 0.472 | 0.487 | 0.453 | 0.445 | 0.597 | 0.528 |
| Auxin | ICA | Indole-3-carboxylic Acid | 4.14 | 7.18 | 6.72 | 2.13 | 4.88 | 3.99 |
| Auxin | IAA | Indole-3-acetic acid | 21.5 | 17.7 | 20.8 | 21.9 | 23.4 | 21.58 |
| Auxin | ICAld | Indole-3-carboxaldehyde | 38.3 | 51.9 | 49.3 | 25.4 | 40.1 | 37.8 |
| Auxin | MEIAA | Methyl indole-3-acetate | 0.915 | 0.616 | 0.892 | 0.727 | 0.798 | 0.681 |
| **Auxin** | **IPA** | **3-Indolepropionic acid** | **95** | **92.2** | **89.6** | **73.8** | **52.8** | **67.1** |
| CTK | DHZR | Dihydrozeatin ribonucleoside | 2.98 | 0.91 | 1.39 | 0.77 | 0.958 | 0.584 |
| CTK | cZR | cis-Zeatin riboside | 0.834 | 0.636 | 0.598 | 0.689 | 0.718 | 0.633 |
| CTK | mTR | meta-Topolin riboside | 0.0611 | 0.083 | 0.096 | 0.0723 | 0.0927 | 0.084 |
| CTK | oT | ortho-Topolin | 0.246 | 0.2 | 0.267 | 0.0926 | 0.189 | 0.134 |
| CTK | BAP | 6-Benzyladenine | 0.0474 | 0.0873 | 0.062 | 0.844 | ND | 0.522 |
| CTK | BAPR | 6-Benzyladenosine | 0.0538 | 0.036 | 0.039 | 0.023 | 0.0286 | 0.026 |
| CTK | KR | Kinetin riboside | 0.205 | 0.179 | 0.207 | 0.14 | 0.117 | 0.151 |
| CTK | oTR | ortho-Topolin riboside | ND | 0.12 | 0.135 | ND | ND | ND |
| CTK | iP9G | N6-Isopentenyl-adenine-9-glucoside | ND | 0.194 | 0.213 | ND | ND | ND |
| CTK | iP7G | N6-Isopentenyl-adenine-7-glucoside | 0.431 | 0.403 | 0.437 | 0.346 | 0.304 | 0.288 |
| CTK | tZOG | trans-Zeatin-O-glucoside | 4.66 | 3.48 | 4.09 | 2.38 | 3.63 | 2.97 |
| CTK | DHZROG | Dihydrozeatin-O-glucoside riboside | 4.85 | 4.07 | 4.57 | 1.97 | 3.39 | 2.85 |
| CTK | cZROG | cis-Zeatin-O-glucoside riboside | 19.9 | 17.3 | 17.6 | 11.3 | 14.1 | 13.8 |
| CTK | BAP9G | N6-Benzyladenine -9-glucoside | 0.255 | ND | 0.271 | 0.112 | 0.161 | 0.134 |
| CTK | K9G | Kinetin-9-glucoside | 1.06 | 0.761 | 0.855 | 0.781 | 0.542 | 0.632 |
| CTK | 2MeScZ | 2-methylthio-cis-zeatin | 0.0867 | 0 | 0.103 | ND | ND | ND |
| CTK | 2MeScZR | 2-Methylthio-cis-zeatin riboside | 1.68 | 1.35 | 1.43 | 0.816 | 1.14 | 0.928 |
| CTK | cZ | cis-Zeatin | ND | 0.11 | 0.095 | ND | ND | ND |
| CTK | DHZ7G | Dihydrozeatin-7-glucoside | 0.828 | 0.605 | 0.692 | 0.734 | 1.46 | 0.957 |
| CTK | DZ | Dihydrozeatin | 0.269 | 0.142 | 0.233 | 0.112 | 0.242 | 0.185 |
| CTK | IP | N6-isopentenyladenine | 0.176 | 0.205 | 0.196 | 0.123 | 0.0788 | 0.099 |
| CTK | IPR | N6-isopentenyladenosine | 17.2 | 14.8 | 16.8 | 13.2 | 12.5 | 12.5 |
| CTK | tZ | trans-Zeatin | 0.712 | 0.211 | 0.577 | 0.25 | 0.325 | 0.296 |
| **CTK** | **tZR** | **trans-Zeatin riboside** | **54** | **35** | **49** | **14.04** | **14.72** | **13.85** |
| ETH | ACC | 1-Aminocyclopropanecarboxylic acid | 38.9 | 27.6 | 34.9 | 31.2 | 37.3 | 36.7 |
| GA | GA15 | Gibberellin A15 | 0.524 | 1.36 | 0.928 | 1.64 | 1.23 | 1.392 |
| GA | GA19 | Gibberellin A19 | 3.02 | 2.17 | 2.59 | 2.95 | 3.45 | 3.09 |
| GA | GA3 | Gibberellin A3 | 7.52 | 5.23 | 5.86 | 7.36 | 7.8 | 6.93 |
| GA | GA53 | Gibberellin A53 | ND | ND | ND | 2.25 | 3.93 | 2.71 |
| GA | GA7 | Gibberellin A7 | ND | 0.182 | 0.093 | ND | ND | ND |
| JA | JA-Val | N-[(-)-Jasmonoyl]-(L)-valine | 1.69 | 2.86 | 1.95 | 1.22 | 2.4 | 1.94 |
| JA | OPC-4 | 3-oxo-2-(2-(Z)-Pentenyl) cyclopentane-1-butyric acid | 44.7 | 68.1 | 59.8 | 29.5 | 37.5 | 30.9 |
| JA | OPC-6 | 3-oxo-2-(2-(Z)-Pentenyl) cyclopentane-1-hexanoic acid | 36.2 | 40.5 | 36.2 | 25.8 | 66.9 | 59.7 |
| JA | H2JA | Dihydrojasmonic acid | 0.385 | 0.515 | 0.397 | 0.455 | 0.496 | 0.458 |
| JA | JA | Jasmonic Acid | 96.9 | 170 | 132 | 89.6 | 115 | 99.6 |
| JA | JA-ILE | Jasmonoyl-L-isoleucine | 23 | 57.1 | 44.3 | 19.9 | 52.6 | 32.06 |
| JA | OPDA | cis(+)-12-Oxophytodienoic acid | 83 | 62 | 75 | 173 | 130 | 144 |
| SA | SA | Salicylic Acid | 129 | 153 | 137 | 152 | 199 | 183 |

ND: Not detect.

Supplemental Table 2 Primer sequences used for RT-qPCR detection

| No. | Genes | Sequence (5’–3’) | GenBank  Accession No. |
| --- | --- | --- | --- |
| 1 | *PpALDH3F1* | F: AACCTCTAGCCATCTATGCCTTCAC | LOC103938024 |
|  |  | R: AGCCACTCTGACCAACACCAC |  |
| 2 | *PpYUCCA2* | F: GCTCATACTCATGCCATTCCCTTC | LOC103962964 |
|  |  | R: GCACTCACCACTGTCCTGTTG |  |
| 3 | *PpCKX3* | F: GCAGGAGTGTTCAAGGACATTGTTC | LOC103944001 |
|  |  | R: CGGTTCATTGGATAAACGAGGACTG |  |
| 4 | *PpActin* | F: CGCTTGTTTCGCTGTGGATT | JN684184 |
|  |  | R: GTTCCCCAACTTTTGCCACC |  |
